# Spheroid Fabrication Using Concave Microwells Enhances the Differentiation Efficacy and Function of Insulin-Producing Cells via Cytoskeletal Changes

**DOI:** 10.3390/cells9122551

**Published:** 2020-11-27

**Authors:** Yu Na Lee, Hye Jin Yi, Hanse Goh, Ji Yoon Park, Sarah Ferber, In Kyong Shim, Song Cheol Kim

**Affiliations:** 1 Asan Medical Center, Asan Institute for Life Sciences, University of Ulsan College of Medicine, Seoul 05505, Korea; ynlee426@naver.com (Y.N.L.); 2hyejinyi@gmail.com (H.J.Y.); standard0130@gmail.com (H.G.); jiyoonpark16@gmail.com (J.Y.P.); 2 Asan Medical Center, Department of Medical Science, Asan Medical Institute of Convergence Science and Technology (AMIST), University of Ulsan College of Medicine, Seoul 05505, Korea; 3 Department of Chemistry, Wesleyan University, Middletown, CT 06457, USA; 4 Regenerative Medicine, Stem Cell and Tissue Engineering Center, Sheba Medical Center, Tel-Hashomer 52621, Israel; sarahferber@gmail.com; 5 Dia-Cure, Acad. Nicolae Cajal Institute of Medical Scientific Research, Titu Maiorescu University, 022328 Bucharest, Romania; 6 Orgenesis Ltd., Ness-Ziona 7403631, Israel; 7 Department of Human Genetics, Sackler School of Medicine, Tel Aviv University, Tel Aviv 6997801, Israel; 8 Asan Medical Center, Department of Surgery, University of Ulsan College of Medicine, Seoul 05505, Korea

**Keywords:** insulin-producing cells, spheroid, three-dimensional culture, concave microwell, diabetes, cytoskeleton changes

## Abstract

Pancreatic islet transplantation is the fundamental treatment for insulin-dependent diabetes; however, donor shortage is a major hurdle in its use as a standard treatment. Accordingly, differentiated insulin-producing cells (DIPCs) are being developed as a new islet source. Differentiation efficiency could be enhanced if the spheroid structure of the natural islets could be recapitulated. Here, we fabricated DIPC spheroids using concave microwells, which enabled large-scale production of spheroids of the desired size. We prepared DIPCs from human liver cells by trans-differentiation using transcription factor gene transduction. Islet-related gene expression and insulin secretion levels were higher in spheroids compared to those in single-cell DIPCs, whereas actin–myosin interactions significantly decreased. We verified actin–myosin-dependent insulin expression in single-cell DIPCs by using actin–myosin interaction inhibitors. Upon transplanting cells into the kidney capsule of diabetic mouse, blood glucose levels decreased to 200 mg/dL in spheroid-transplanted mice but not in single cell-transplanted mice. Spheroid-transplanted mice showed high engraftment efficiency in in vivo fluorescence imaging. These results demonstrated that spheroids fabricated using concave microwells enhanced the engraftment and functions of DIPCs via actin–myosin-mediated cytoskeletal changes. Our strategy potentially extends the clinical application of DIPCs for improved differentiation, glycemic control, and transplantation efficiency of islets.

## 1. Introduction

Type 1 diabetes mellitus (T1DM) is characterized by insulin deficiency that results from the destruction of pancreatic islets, a phenomenon that is the result of an autoimmune response (or other unknown causes). Although exogeneous insulin is generally used for T1DM treatment, it is difficult to prevent the long-term side effects of insulin that arise from the inability to precisely respond to the blood glucose concentration [1,2,3]. Pancreas and pancreatic islet transplantation are the fundamental therapies for insulin-dependent diabetes. Pancreatic islets are transplanted by trans-hepatic injection via catheterization of the portal vein; this is simpler and safer than whole-pancreas transplantation. Due to the use of efficacious anti-rejection therapies and development of transplantation techniques, the long-term insulin-independence rate associated with clinical islet transplantation has improved [4,5]; however, donor shortage remains a major hurdle [6]. Insulin-producing cells (DIPCs)—differentiated from stem cells or adult cells—are being developed as a new islet source [7]. Embryonic stem cells or induced pluripotent stem cells are generally used as cell sources. Additionally, somatic or adult stem cells with a non-pancreatic origin have been transdifferentiated into DIPCs directly through gene modifications [8,9,10,11]. Although these cells showed insulin production and genetic changes in vitro, they could not recapitulate physiological function in clinical applications.

The three-dimensional (3-D) structure of cell–cell interactions among islets is crucial for maintaining their physiological function. Separating pancreatic islets into individual cells results in a decrease in insulin levels because cell–cell interactions are involved in the functioning of pancreatic islets and maturation of DIPCs [12]. Recently, studies have shown that recapitulation of the 3-D structure of natural islets allows the DIPCs to maintain cell–cell interactions during differentiation and maturation, an outcome that can overcome the low trans-differentiation efficiency associated with normal DIPCs [13,14]. Numerous 3-D culture techniques have been investigated for stem cell differentiation. Generally, cells at a high density are suspended under continuous stirring or shaking or embedded into hydrogel to produce cell clumps and aggregates [15,16,17]. Although these techniques have been successful, their ability to mass produce spheroids of the desired size is limited. Suspension culture techniques cannot control spheroid size and shape while achieving cell agglomeration, which can lead to necrosis at the center of spheroids or uncontrolled differentiation. Shear force-induced apoptosis in the suspension method is also common [15]. For clinical applications, is important to fabricate spheroids of the desired size for long-term culture and mass production. Recently, several studies have shown the production of cancer cell spheroids using microchips and have demonstrated their use in drug screening [18,19,20] and islet/DIPC reaggregation [21,22]. However, whether the 3-D spheroid structure of natural islets could enhance the differentiation or function of DIPC has not been clarified. Here, we aimed to establish a technique to mass produce DIPC spheroids of the desired size using concave microwells and to find a key factor governing improvement in spheroid-induced DIPC function.

## 2. Materials and Methods

### 2.1. Cell Isolation

Adult human liver cells were isolated from human liver tissues and cultured as previously described [11,23]. This study was approved by and followed the guidelines of the Institutional Review Board of Asan Medical Center (IRB number: 2014–1182, Seoul, Korea). Liver tissues were digested using collagenase type I (Worthington Biochemical, Lakewood, NJ, USA). Cells were washed and seeded on fibronectin-coated plates (3 μg/cm^2^, Sigma Aldrich, St. Louis, MO, USA). Cells were cultured in low-glucose Dulbecco’s modified Eagle’s medium supplemented with 10% fetal bovine serum, 1% antibiotic-antimycotic solution, and Glutamax (Life Technologies, Grand Island, NY, USA) at 37 °C. Upon reaching 85% confluence, cells were split (1:3) using trypsin-EDTA. After propagation, liver cells at passage 5–7 were used for experiments.

### 2.2. Trans-Differentiation of Liver Cells into DIPCs

To induce trans-differentiation, transcription factors regulating β-cell function and physiology (PDX1, NEUROD, and MAFA) were transduced into liver cells using adenoviral expression vectors. Ad-CMV-hPDX1 (adenovirus-cytomegalovirus-PDX1), Ad-CMV-hNEUROD1, and Ad-CMV-hMAFA were obtained from Vector Biolabs (Malvern, PA, USA). The multiplicity of infection (MOI) of the viruses were Ad-CMV-PDX1, 500; Ad-CMV-NEUROD1, 250; and Ad-CMV-MAFA, 50. Optimal MOI was determined based on cell survival, insulin promotor assay results, and insulin gene expression in a previous study. Human liver cells were transduced with Ad-CMV-PDX1 and Ad-NEUROD1 for 2 days, the medium was changed, and Ad-MAFA was transduced for 3 days. The expression of the transduced transcription factors was confirmed using immunostaining of 2-D-cultured DIPCs and DIPC spheroids 5 days after initial virus treatment. To induce DIPC trans-differentiation, liver cells were cultured in media supplemented with 10 mM nicotinamide (Sigma Aldrich), 20 ng/mL epidermal growth factor (PeproTech, Rocky Hill, NJ, USA), and 5 nM exendin-4 (Sigma Aldrich).

### 2.3. DIPC Spheroid Formation

**Figure 1:**
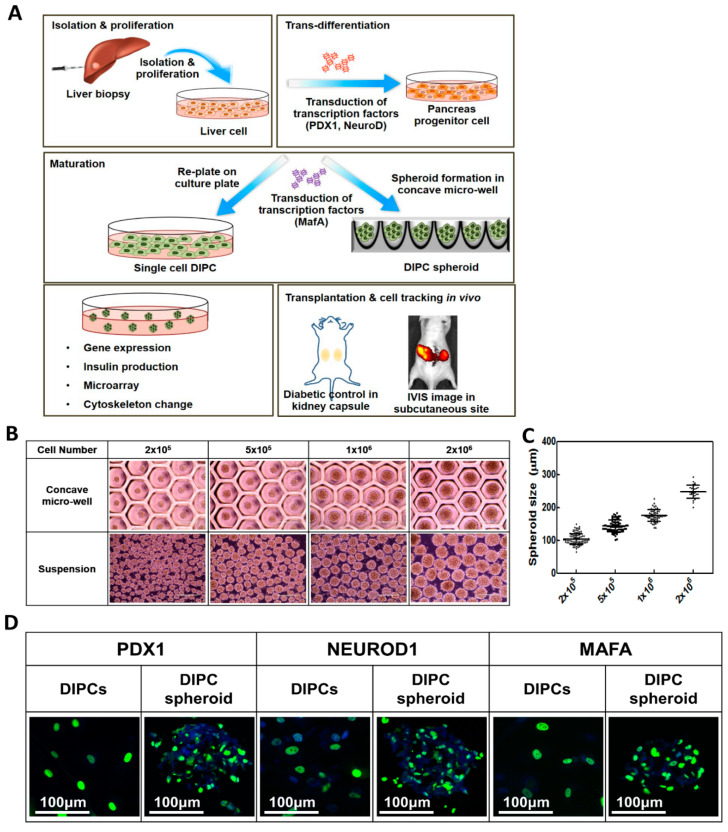
Fabrication of DIPCs and DIPC spheroids. (**A**) Schematic diagram of the experimental procedure. Firstly, adult human liver cells were isolated from liver and trans-differentiated to DIPCs using three pancreatic transcription factors, PDX1, NEUROD1, and MAFA, which were carried by recombinant adenoviruses and sequentially supplemented. To prepare spheroids, cells were seeded in concave microwells. After 24 h, cells aggregated to form spheroids, which were collected by flushing the medium and cultured in suspension culture plates. To compare single DIPCs, cells were cultured in conventional 2-D plates. At day 5, DIPCs and DIPC spheroids were harvested for in vitro assays and in vivo transplanted. (**B**) Images and (**C**) size distribution of DIPC spheroids in concave microwells and suspension. (**D**) Ectopic gene expression (PDX1, NeuroD, and MAFA) in DIPCs and spheroids.

The scheme of this study is summarized in Figure 1A. To prepare spheroids, cells were seeded on commercially available concave microwells (StemFIT 3D H853400; Microfit, Seoul, Korea) coated with 3% (*w*/*v*) bovine serum albumin (BSA; Cellnest, NJ, USA) to prevent cell attachment. After 24 h, the cells aggregated to form spheroids, which were then collected by flushing the medium and cultured in suspension culture plates. Before preparing the 3-D spheroids, we selected the spheroid size based on the cell number. Single DIPCs were seeded in concave microwells at densities of 2 × 10^5^, 5 × 10^5^, 1 × 10^6^, and 2 × 10^6^ cells/well. The spheroid size was confirmed by microscopy in suspension culture. To make IPC spheroids using the suspension culture technique as a control, 2 × 10^6^ cells in 12 mL of media were seeded on a suspension culture plate with a 100-mm diameter and incubated with 50 rpm in the rotating incubator (CO_2_-resistant shakers, ThermoFisher Scientific, Cambridge, UK). After 24 h, the cells were aggregated to form spheroids and statistically cultured under the same conditions of IPC spheroids made by a concave microwell. A cell viability assay of spheroids with different techniques was done on day 3 after spheroid formation, using live/dead staining with fluorescein diacetate/propidium iodide (FDA/PI).

It was important to ensure that the genes coding for the transcription factors were effectively transduced into all cells in the 3-D spheroids as this transduction would result in the trans-differentiation of liver cells. Thus, various conditions for seeding cells into concave microwells were optimized. After transducing the liver cells with green fluorescent protein (GFP, MOI 200), GFP expression was assessed by flow cytometry in various culture conditions in concave microwells.

### 2.4. Flow Cytometry

To determine GFP expression, liver cells at passage 6 transduced using Ad-CMV-GFP (MOI 200) in 2-D culture dishes or spheroids in concave microwells were harvested using trypsin. Cells were analyzed on FACSCalibur (BD Biosciences, Franklin Lakes, NJ, USA).

### 2.5. Immunocytochemistry of DIPCs and DIPC Spheroids

Five days after gene transduction, the expression of insulin and glucagon was checked in DIPCs. Guinea pig anti-insulin and mouse anti-glucagon (1:1000; Abcam, Cambridge, UK) were used as primary. To determine the expression of transduced pancreatic endocrine transcription factors, we used rabbit anti-PDX1, mouse anti-NEUROD1, and rabbit anti-MAFA (1:200; Abcam, Cambridge, MA, USA). Samples were incubated overnight with primary antibodies at 4 °C. For secondary fluorescence labeling, cells were incubated with anti-guinea pig IgG Alexa Fluor 647 (1:200; Abcam, MA, USA), anti-rabbit IgG Alexa Fluor 488 (1:200; Thermo Fisher Scientific, Waltham, MA, USA), and anti-mouse IgG Alexa Fluor 488 (1:200; Thermo Fisher Scientific). Finally, cells were mounted using prolong gold antifade mountant containing DAPI (4′,6-Diamidino-2-phenylindole) (Life Technologies, Frederick, MD, USA). The slides were visualized under an EVOS FL auto cell-imaging system (Thermo Fisher Scientific). For DIPC spheroid staining, spheroids were harvested and fixed with 4% paraformaldehyde, processed in 30% sucrose for 3 days, and then embedded in optimum cutting temperature compound (Tissue-Tek; Sakura Finetek, Tokyo, Japan). Cryostat sections (6 μm) were sliced, and the subsequent process was the same as that for DIPCs.

### 2.6. Electron Microscopy

To analyze the granular ultrastructure, the cells were fixed with 1% glutaraldehyde and 1% paraformaldehyde in 0.1 M sodium cacodylate buffer (pH 7.2) at 4 °C. Specimens were then fixed in 2% osmium tetraoxide for 60 min at 4 °C. Dehydration of the fixed samples was performed and the samples were transferred to Lowicryl resin (Polyscience, Niles, IL, USA). Samples were sectioned (60 nm) with an ultramicrotome (UltracutUCT, Leica, Wetzlar, Germany) and collected on nickel grids. Post-embedding immunogold labeling was performed for insulin and glucagon labeling using the rabbit monoclonal anti-insulin antibody (Abcam; ab181547), mouse monoclonal anti-glucagon antibody (Abcam; ab10988), 5-nm colloidal gold conjugated to goat anti-rabbit IgG secondary antibodies (Sigma), and 9–11-nm colloidal gold conjugated to goat anti-mouse IgG secondary antibodies (Sigma). Following immunogold labeling, the sections were double stained with 2% uranyl acetate for 20 min and lead citrate for 10 min. The sections were then viewed under a transmission electron microscope.

### 2.7. Quantitative Real-Time PCR (qPCR)

Total RNA was extracted from DIPCs and DIPC spheroids on day 5 using TRIzol (Thermo Fisher Scientific) according to the manufacturer’s instructions. Real-time PCR was performed using LightCycler 480 SYBR Green I Master mix in a LightCycler 480 II real-time thermal cycler (Roche Applied Science, Mannheim, Germany). The primer sets used for amplifying pancreatic endocrine-specific genes and cadherin are listed in Supplementary Materials Table S1. Gene expression was normalized to that of GAPDH (housekeeping gene), and relative quantification was performed using the delta Ct method. Statistical analyses were performed using *t*-tests and reported in all figures.

### 2.8. Insulin and C-Peptide Release

Insulin and C-peptide secretion was measured in the culture medium of DIPCs and DIPC spheroids by static incubation for 2 days in differentiation culture medium containing 5.5 mM glucose. Additionally, DIPCs and spheroids were washed and lysed using RIPA lysis buffer to detect intracellular insulin contents [24]. Prior to glucose stimulation of cells, residual insulin released from cells was removed by incubating in serum- and glucose-free Krebs buffer. The tubes were incubated at 37 °C for 1 h with shaking; then, the medium was completely removed by centrifugation and replaced with Krebs buffer supplemented with 3 mM glucose for 1 h. After collecting the medium, the cells were incubated with Krebs buffer containing 30 mM glucose for 1 h. Supernatants were collected. The stimulation index was calculated by dividing the insulin concentration of the supernatant in response to the 30 mM glucose incubation by that of the supernatant from the 3 mM glucose incubation [25]. Insulin and C-peptide levels were measured using a commercial ultrasensitive insulin ELISA kit (ALPCO, Salem, NH, USA) and ultrasensitive C-peptide ELISA kit (Mercodia, Uppsala, Sweden), respectively.

### 2.9. Microarray Analysis of DIPCs and DIPC Spheroids

Gene expression data for DIPCs and DIPC spheroids were obtained using Agilent SurePrint G3 Human GE 8X60K, V3 Microarrays (Agilent Technologies, Santa Clara, CA, USA). RNA labeling and hybridization were performed using the Agilent One-Color Microarray-Based Gene Expression Analysis protocol (Agilent Technologies, V 6.5, 2010). The slides were incubated for 17 h at 65 °C in an Agilent hybridization oven and then washed at RT using the abovementioned Agilent protocol. The hybridized array was immediately scanned using an Agilent Microarray Scanner D (Agilent Technologies). Raw data were extracted using the Agilent Feature Extraction Software (v11.0.1.1). Array probes that had Flag A in samples were filtered out. The selected gProcessedSignal value was log-transformed and normalized by the quantile method. Significance of the expression data was determined based on fold change. All data analyses and visualization of differentially expressed genes were conducted using R 3.1.2 (www.r-project.org).

### 2.10. Treatment of Inhibitors for Actin–Myosin Interactions

To evaluate the effect of actin–myosin interactions on differentiation, the gene expression profile of DIPCs treated with 10 μM blebbistatin (Sigma Aldrich) or 10 nM Y-27632 (Sigma Aldrich) was compared with that of untreated DIPCs and spheroids. For each group, experimental samples were treated with drugs, whereas controls were treated with an equal volume of dimethyl sulfoxide. After treatment with the supplemented medium for 3 days (days 2–5), the samples were assessed for cytoskeletal staining and gene expression. F-actin was stained using Alexa-488 phalloidin.

### 2.11. Cell Labeling and Imaging Ex Vivo and In Vivo

DIPCs were labeled with Qdot 800 (Qtracker 800; Molecular Probes, Inc., Eugene, OR, USA). After labeling with Qdot 800, cells were trans-differentiated for 5 days and transplanted into the subcutaneous site. In vivo optical imaging was performed using a Spectrum in vivo imaging system (IVIS) (Caliper Life Science, Inc., Waltham, MA, USA) at 430 nm excitation and 800 nm emission. A grayscale body image was collected and overlaid with a pseudo-color image representing the spatial distribution of the detected photons. Fluorescence was quantified as the sum of all detected photon counts per second within a constant region of interest for each transplant site using Living Image software (Xenogen).

### 2.12. DIPCs and DIPC Spheroid Transplantation

Animal experiments were approved by the Institutional Animal Care and Use Committee of Asan Institute for Life Sciences. Male 8-week-old BALB/c nude mice were treated with 180 mg/kg streptozotocin (Santa Cruz Biotechnology, Santa Cruz, TX, USA) dissolved in 0.1 M citrate buffer, pH 4.5. Blood glucose levels were monitored using a Codefree blood glucose monitoring system (SD Biosensor, Suwon, Korea). Diabetes was induced in mice when blood glucose levels were over 300 mg/dL for three consecutive days. DIPCs and DIPC spheroids were harvested. The cell number per spheroid was calculated (10^3^/spheroid). Cells (2 × 10^6^ DIPCs/tube or 2 × 10^3^ spheroids/tube) were transferred to PE50 tubing. After anesthetizing the mice with isoflurane, we exposed the kidney and slowly injected the cells in the tubing under the kidney capsule using a Hamilton syringe. After removing the tubing, the nick in the kidney capsule was carefully closed, and the kidney was gently replaced inside the peritoneum. After cell transplantation, the blood glucose levels and body weights of mice were monitored.

### 2.13. Histological Analysis

After mice were euthanized on days 3 and 14, the kidney was removed and fixed in 10% formalin. A paraffin block was prepared with the fixed tissue and cut into 4-μm sections. Samples were deparaffinized, dehydrated, and subjected to staining with hematoxylin and eosin (Sigma Aldrich). Immunohistochemistry was performed using primary antibodies for rabbit anti-PDX1 and rabbit anti-insulin (dilution 1:1000, Abcam, Cambridge, UK). An automated slide preparation system (Benchmark XT; Ventana Medical Systems Inc, Tucson, AZ, USA) with an OptiView DAB Detection Kit (Ventana Medical Systems) was used for immunohistochemistry.

### 2.14. Statistical Analysis

The data are presented as the mean ± standard deviation with the number of samples indicated in the figure legends. The significance of the differences between multiple groups was analyzed by one-way analysis of variance; the differences between the means were compared with Tukey’s post-hoc test. The significance of the difference between the two groups was analyzed using Student’s *t*-test. *p* < 0.05 indicated a significant difference.

### 2.15. Ethics Approval and Consent to Participate

This animal study was reviewed and approved by the Institutional Animal Care and Use Committee (IACUC No. 2015-12-123) of Asan Institute for Life Sciences. The committee abides by the Institute of Laboratory Animal Resources (ILAR) guidelines. All experiment protocols of human liver cells isolation were carried out according to the guidelines and with the approval of the Institutional Review Board of Asan Medical Center (IRB number: 2014–1182, Seoul, Korea). We obtained written informed consent from all patients who participated in this study.

## 3. Results

### 3.1. Spheroid Size Distribution

Figure 1B shows spheroids of DIPC spheroid in the concave microwell and suspension. The sizes of DIPC spheroids were measured in suspension. Micrographs of spheroids were taken in randomly selected fields per well 1 day after culture. DIPC spheroids of 2 × 10^5^, 5 × 10^5^, 1 × 10^6^, and 2 × 10^6^ cells/mL/mold had sizes of 104.3 ± 16.05, 142.5 ± 18.07, 175.8 ± 17.95, and 247.7 ± 20.59 μm, respectively. The spheroid size consistently increased as the cell number increased, and an association was found with DIPCs (Pearson’s coefficient: 0.922). DIPC spheroids containing 10^6^ cells/mold were used for the functional study due to the similar size of ideal pancreatic islets. Supplementary Materials Figure S2A,B shows the morphology and size distribution of IPC spheroids made by the suspension culture technique using a shaking incubator as a control. Both IPC spheroids made by suspension culture and by the concave microwell showed a spherical shape. The average diameter of IPC spheroids from suspension culture is 152.88 + 83.98, which is slightly less than that of those obtained from concave microwells (175.62 + 16.81). The size distribution of spheroids (10^6^ cells per well) made from concave microwells was relatively uniform, but spheroids made from suspension cultures had a very wide size distribution, and some very large aggregates formed.

### 3.2. Ectopic Gene Expression of Transduced Transcription Factors

To optimize the transduced gene expression, we used GFP. Ectopic Ad-GFP expression in liver cells was confirmed by fluorescence microscopy and flow cytometry (Supplementary Materials Figure S1). Figure S1 shows the scheme of gene treatments during spheroid formation. When spheroids had already formed in the wells, only surface-level cells (36.0 ± 11.1% of cells) were transduced and expressed GFP. However, when the medium and cells were simultaneously mixed with adenoviral vectors in the microwells, >80% cells were transduced and sufficiently expressed ectopic genes. Upon treating 2-D culture dishes with Ad-GFP, >97% of cells expressed genes. Consequently, we ectopically introduced transcription factor-coding genes. Firstly, PDX1 and NEUROD1 were transduced in liver cells for 2 days, followed by MAFA for maturation for 3 days. Based on the GFP expression results, we treated 2-D culture plates with PDX1 and NEUROD1 and then mixed MAFA into microwells. Ectopic gene expression of PDX1, NEUROD1, or MAFA in DIPCs and DIPC spheroids was confirmed by immunohistochemistry (Figure 1D). PDX1 and NEUROD1 were expressed in most cells, while MAFA was partially expressed. There was no significant difference between DIPCs and DIPC spheroids.

### 3.3. Gene Expression in DIPCs and DIPC Spheroids

**Figure 2:**
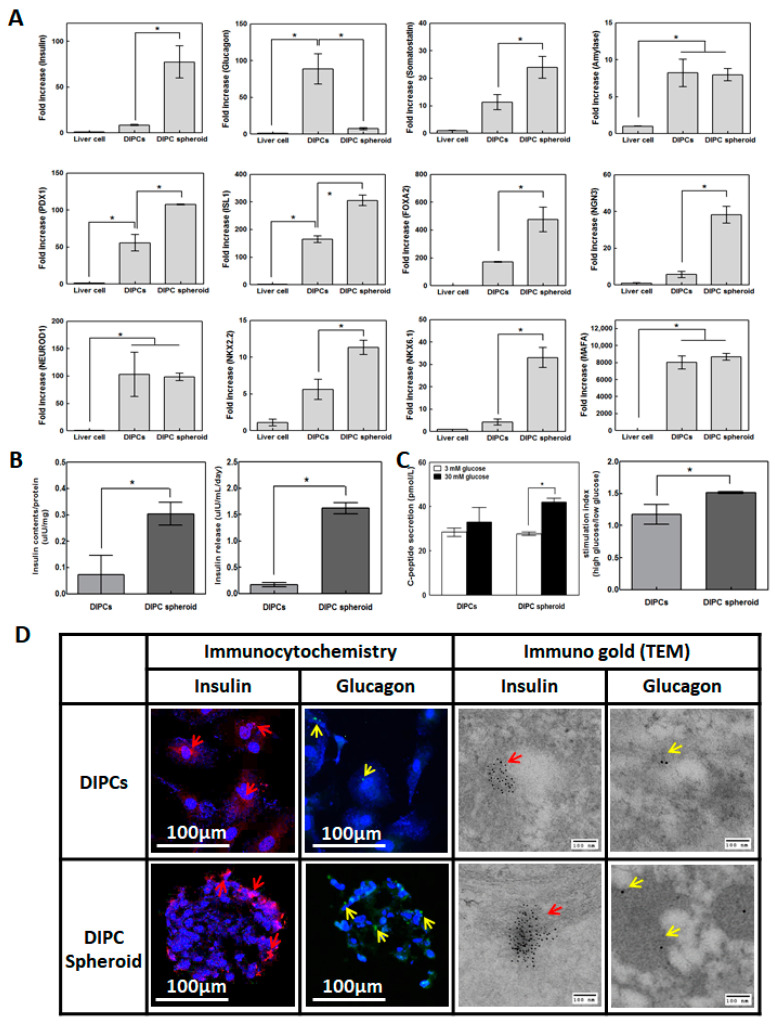
Trans-differentiation efficacy of DIPCs and DIPC spheroids. (**A**) The gene expression of insulin, glucagon, somatostatin, amylase, and pancreas-specific transcription factors (*n* = 4). (**B**) Insulin contents of and release from DIPCs and spheroids (*n* = 4). Spheroids showed higher insulin production than DIPCs. * indicates a significant difference between two groups (*p* < 0.05). (**C**) The glucose-stimulated insulin secretion index was calculated by dividing the c-peptide concentration of the supernatant of cells incubated with 30 mM glucose by that of cells incubated with 3 mM glucose (*n* = 4). (**D**) Representative insulin and glucagon staining using immunocytochemistry and TEM in DIPCs and DIPC spheroids. Red: insulin; green: glucagon; blue: nuclei; Original magnifications: ×100. The red arrow indicates an insulin immunoGold aggregate (9–11 nm); the white arrow indicates a glucagon immunoGold aggregate dot (5 nm).

DIPC differentiation in different culture conditions was compared by analyzing the gene expression profiles of endocrine hormones and pancreatic transcription at day 5. DIPCs and DIPC spheroids showed higher expression of insulin, glucagon, somatostatin, amylase, and pancreas-specific transcription factors, including PDX1, ISL1, FOXA2, NGN3, NEUROD1, NKX2.2, NKX 6.1, and MAFA, than control liver cells (Figure 2A). Particularly, insulin mRNA levels in DIPC spheroid were significantly higher than those in DIPCs on culture plates, whereas glucagon mRNA was not activated in DIPC spheroids. Similarly, pancreatic transcription factors related to β-cell differentiation were significantly higher in DIPC spheroids than in single-cell culture (*p* < 0.05). Gene expression and insulin content on IPC spheroids compared with liver cells and IPCs are shown in Figure S2C,D. Both IPCs made by suspension culture and by concave microwell showed similar endocrine gene expression levels and were higher than IPCs. However, the insulin content of IPC spheroids made by suspension culture is higher than that of IPCs but lower than that of microwell IPC spheroids.

### 3.4. Insulin and Glucagon Protein Expression

To confirm insulin production in DIPCs and DIPC spheroids, insulin contents and secretion were measured at day 5 after the initial viral treatment. Insulin contents and secretion were significantly increased in DIPC spheroids compared to those in DIPCs (*p* < 0.05, Figure 2B). To further examine physiological insulin secretion, we evaluated C-peptide secretion at two different glucose concentrations (3 and 30 mM). The secretion in DIPC spheroids showed a significantly higher glucose-sensing response (*p* < 0.05, Figure 2C).

Immunohistochemistry and transmission electron microscopy were used to confirm insulin and glucagon production in DIPCs and DIPC spheroids. Insulin- and glucagon-positive cells were stained (Figure 2D). Insulin aggregates were observed in DIPC spheroids, while small amounts of glucagon were spread throughout the cytosol. More clearly, insulin-positive aggregates were observed in DIPC spheroids than in DIPCs.

### 3.5. DIPCs and DIPC Spheroid Transplantation

**Figure 3:**
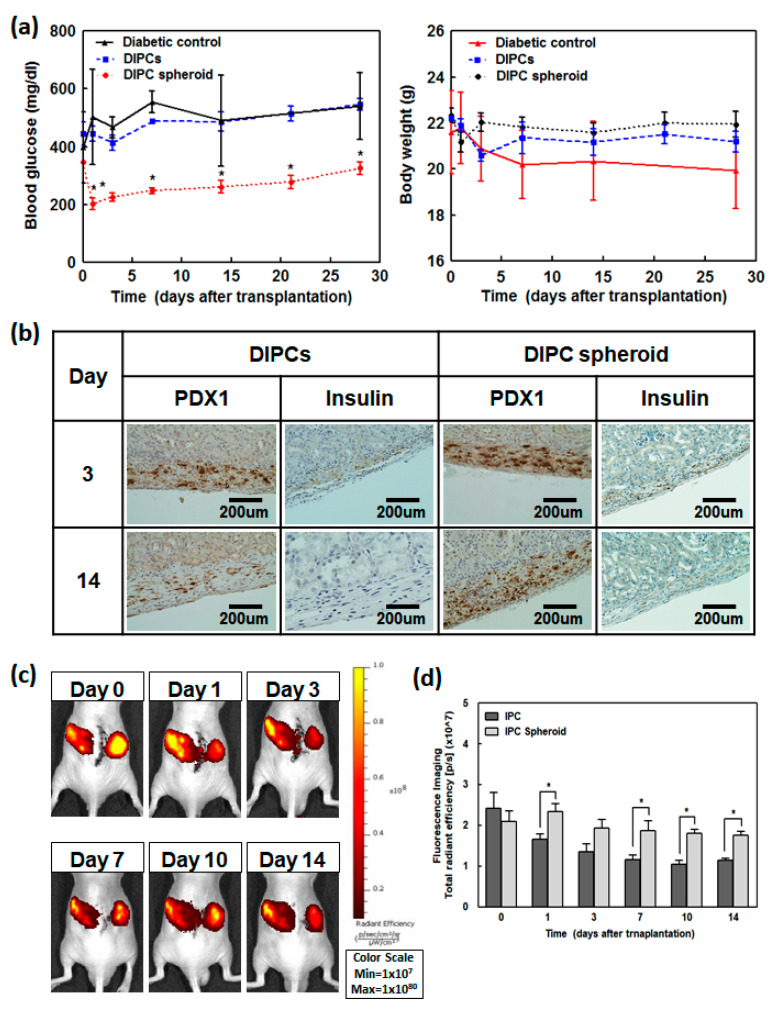
Therapeutic effects of DIPCs and DIPC spheroid transplantation in diabetic nude mice. Insulin-dependent diabetes was induced in mice using streptozotocin. On day 0, mice were transplanted with DIPCs or DIPC spheroids in the kidney capsule. Untreated diabetic mice were used as a negative control. (**A**) Non-fasting blood glucose levels and body weights were measured at different time points in diabetic control, DIPCs-, or DIPC spheroids-transplanted mice (*n* = 4). The blood glucose level of the DIPC spheroids group decreased to 200 mg/dL after transplantation. Single-DIPC-transplanted mice revealed an increase in blood glucose for 4 weeks with no decrease. * indicates a significant reduction in blood glucose compared to DIPC; *p* < 0.05. (**B**) Immunohistochemical analyses of PDX1 and insulin in the liver 3 and 14 days after transplantation. Arrows indicate positive cell staining. Original magnifications: ×200 (PDX1); ×400 (insulin). Scale bars: 200 µm (PDX1); 100 µm (insulin). (**C**) Comparison of the transplantation efficiency between transplantation of DIPCs and spheroids in the subcutaneous site of mice. The images of transplanted DIPCs and spheroids were examined with an in vivo imaging system. DIPCs were optically imaged by labeling with Qdot 800 in vivo. (**D**) Quantification of the NIR signals of transplanted cells is expressed as total radiant efficiency (p/s) (*n* = 4). * indicates a significant difference between two groups (*p* < 0.05).

Diabetes was induced by administering 180 mg/kg streptozotocin to immunodeficient nude mice to evaluate the blood glucose control of DIPCs. DIPCs and DIPC spheroids (2 × 10^6^ cells) were transplanted into the kidney capsules of diabetic nude mice. Blood glucose and body weight were evaluated for 4 weeks after transplantation. The blood glucose level of the DIPC spheroid group decreased to 200 mg/dL after transplantation but then gradually increased for 4 weeks. Single-DIPC-transplanted mice revealed an increase in blood glucose for 4 weeks with no decrease. Body weights decreased for diabetic mice without transplantation but remained stable for 4 weeks in DIPCs- or DIPC spheroid-transplanted mice (Figure 3A).

### 3.6. Histological and Immunohistochemical Analyses of Mouse Kidney Capsules with Transplantation

Three and fourteen days after DIPCs and DIPC spheroid transplantation, kidneys were harvested to confirm transplantation by immunohistochemistry. Three days post-transplantation, PDX1-stained cells were found at the transplantation site in both DIPCs- and DIPC spheroid-transplanted rats, some of which were insulin-positive cells. Two weeks post-transplantation, most cells disappeared in the DIPCs group; however, in the DIPC spheroid group, most cells remained, and some showed insulin expression (Figure 3B).

### 3.7. Cell Tracking Using an In Vivo Imaging System (IVIS)

To confirm survival after cell transplantation, cells were labeled with Qdot 800, and fluorescence images were obtained using an IVIS. To obtain non-invasive fluorescence images, DIPCs and DIPC spheroids were implanted on both sides of the subcutaneous site. Mice were imaged at different times after transplantation (Figure 3C). The near-infrared (NIR) intensity was confirmed in transplanted animals through an IVIS image analysis program (Figure 3D). Immediately after transplantation, DIPCs- and DIPC spheroid-transplanted mice showed similar NIR intensities; however, in DIPCs-transplanted animals, the NIR intensity slowly decreased after transplantation, and more than 50% of dots decreased after 1-week post-transplantation. In DIPC spheroid-transplanted mice, a significantly large number of cells survived compared to those with DIPCs.

### 3.8. Mechanistic Analysis of β-Cell Differentiation of DIPC and DIPC Spheroid Cultures

**Figure 4:**
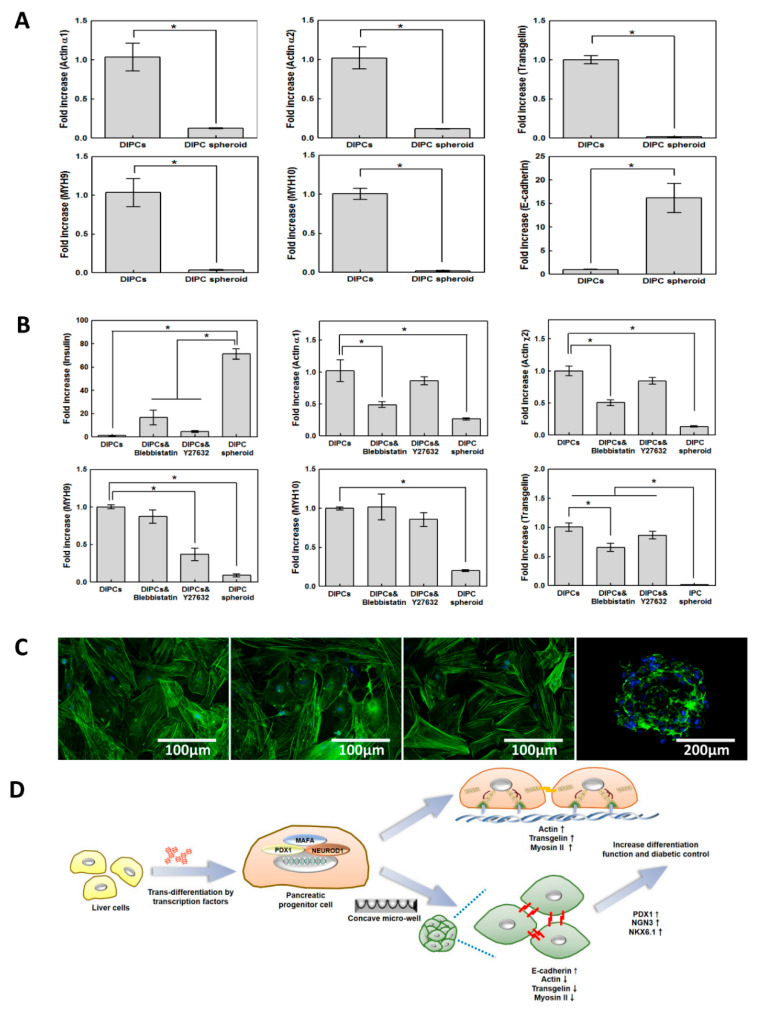
Mechanistic analysis of β-cell differentiation of DIPC and DIPC spheroid cultures (**A**) Comparison of cytoskeletal and E-cadherin gene expression in DIPCs and DIPC spheroids (*n* = 4). (**B**) The effect of treatment with actin and myosin inhibitors on DIPC differentiation (*n* = 4). The gene expression of actin, myosin, and insulin is compared among DIPCs, treated DIPCs, and DIPC spheroids. DIPCs treated with both drugs showed increased insulin gene expression compared to untreated DIPCs, and blebbistatin-treated DIPCs had higher induction of differentiation. * indicates a significant difference between two groups (*p* < 0.05). (**C**) Images of actin in DIPC, treated DIPC, and spheroid groups. Alexa-488 phalloidin was used to visualize F-actin (green); DAPI was used as a nuclear stain (cyan). (**D**) Illustration of the mechanism by which spheroid formation affects insulin production. DIPC spheroid formation reduces actin–myosin interactions and increases cell–cell interactions (E-cadherin), which induce insulin and pancreatic transcription factor expression.

To assess differences in the cytoskeleton mechanism between DIPC and DIPC spheroid systems, we evaluated genes that were differentially upregulated or downregulated in DIPCs and DIPC spheroids using microarray analysis (Supplementary Materials Tables S2 and S3). mRNAs were harvested from DIPCs and DIPC spheroids and independently hybridized to Agilent SurePrint G3 Human GE 8 × 60 K oligonucleotide microarrays. After bioinformatic and gene ontology analyses, genes with significant alterations in expression were selected. Among the downregulated genes in DIPC spheroids, actin and myosin showed significant differences. To validate the microarray data, the expression of actin, TAGLN (an actin-binding protein expressed by TAGLN), and myosin heavy chain was quantified by PCR (Figure 4A). The qPCR data revealed good concordance with the microarray data. To confirm the effect of cytoskeleton interactions on endocrine induction, we treated DIPCs with cytoskeletal dynamic inhibitors. Blebbistatin decreases actin gene expression and actin polymerization, whereas Y-27632 decreases myosin gene expression (Figure 4B). These changes were confirmed using actin cytoskeleton staining (Figure 4C). DIPCs treated with both drugs showed increased insulin gene expression compared to untreated DIPCs, and blebbistatin-treated DIPCs had higher induction of differentiation.

## 4. Discussion

Although pancreatic islet transplantation can be a fundamental treatment for insulin-dependent diabetes, the lack of sources and low transplantation efficiency are major hurdles to its use as a common therapy [6]. Therefore, numerous efforts have attempted to develop new DIPC sources from different cells, including embryonic stem cells, induced pluripotent stem cells, adult stem cells, and somatic cells [26,27,28]. Particularly, adult stem cells and somatic cells are most suited for clinical applications, as they are safe and easy to obtain. However, compared with pluripotent stem cells, adult cells are inefficient at differentiating into β-cells. Various growth factors have been discovered, and 3-D cell culture techniques are being studied to improve the function of DIPCs [29,30]. Forming a 3-D spheroid structure similar to that of natural pancreatic islets is crucial for improving the differentiation efficiency and physiological function of DIPCs. To apply these techniques clinically, it is necessary to mass produce spheroids of the desired shape and size. Additionally, the 3-D structure can be widely used to improve differentiation in cell therapy products. However, whether 3-D spheroid structure of a natural islet size could enhance the differentiation or function of DIPCs has not been revealed. Here, DIPCs of a desired size and shape were fabricated on a large scale using concave microwells and their differentiation and function were evaluated in vitro and in vivo. We further confirmed cytoskeleton changes associated with the actin–myosin system.

Generally, 3-D spheroids are formed in suspension, non-adherent surface, hanging drop, or microfluidic cultures. Suspension culturing allows media to flow, enabling natural spheroid formation. However, this method cannot form uniform-sized spheroids, and shear stress may be applied [31,32]. Necrosis may occur in larger 3-D aggregates due to a poor supply of oxygen and nutrients [33]. In another method, spheroids are formed on non-adherent surfaces that prevent cell adhesion. Although this method is simple, it cannot form uniform-sized spheroids in the liquid overlay technique. Meanwhile, non-adherent-surface microarray wells can enable manufacturing of uniform spheroids, but long-term culturing is difficult [34]. In hanging drop cultures, spheroids cluster in each droplet using gravity. This allows the spheroid size and composition to be controlled; however, the medium exchange process is inefficient. Microfluidic devices can control spheroid size, quickly form spheroids, and allow easy medium exchange [32]. Additionally, experiments can be conducted within a single device. However, cell collection is difficult [35]. Herein, DIPC spheroids were fabricated in PDMS-based concave microwells, which can produce large-scale uniform spheroids. Additionally, seeded cells could easily clump within 1 day. This method can solve the problems posed by other methods, including uniformity, apoptosis, and shear stress. Recently, several studies have been reported on cell culture using customized microwells and 3-D cultures, and such studies are likely to accelerate the development of cell therapy in clinical practice. Tran R et al. used the micro patterning cells in adherent microwells [13]; Pettinato G et al. used round-bottom microwells using nan-cell-adhesive hydrogels in hiPSCs [22]; and Forget A et al. used the specific functionalized poly(dimethylsilosane) (PMDS) petri dish microwells using the insulin-producing immortalized beta cell line MIN6 [36]. Fennema et al. demonstrated that spheroid culture can be used as a tool for creating 3-D complex tissues [34]. Xu B et al. used Matrigel 3-D culture to promote the differentiation of human dental pulp mesenchymal stem cells into insulin-producing cells [29]. Sasaki et al. developed a maskless photolithography device that enables simple mass preparation of size-controlled ESC aggregates for preparation of ESC-derived differentiated cells and high-throughput assay [37]. In terms of differentiated endocrine pancreatic cells from stem cells, a few studies have reported use of microwells. Lee et al. reported a spheroidal 3-D co-culture model of primary rat pancreatic islets and hepatocytes with uniform size and shape using concave microwells [38]. However, growing patterns and characteristics of primary islet cells differ greatly compared to DIPCs. Our study showed the effectiveness of a customized concave-shaped microwell to fabricate the DIPC and confirmed the mechanism of action. Besides, the concave microwells used in this study showed advantages in their simplicity and reliability for clinical application. Additionally, our simple non-adherent concave microwell has the following advantages over other microwell products. First a large amount of cell spheroids of uniform size can be obtained with high reliability. Second, it is made of FDA-approved silicon material, so there is no cytotoxicity, and pure cell culture is possible compared to other products based on material treatment. Unlike plastic products, PDMS microwell has a porous structure and the cells are slightly attached on the surface. When media is changed, it is not affected by media flow, so it can be used for long-term culture without loss of cells. In most cases, cell spheroids can be obtained without a special coating process. Lastly, because it is transparent, it shows good transmittance when observed under a microscope. Many cells can be observed accurately and reliably at once.

Consequently, the concave microwell method could produce spheroids using cells with low initial seeding density, low cell expansion, and low cell volume [15]. We found that the morphological size of spheroids depends on the cell number and that multitudinous spheroids could be obtained using concave microwells. In all conditions, the standard deviation of the spheroid size was 10%, indicating a uniform size distribution. We used DIPC spheroids formed with 10^6^ cells/mold, which were of a similar size to ideal pancreatic islets [39]. Equal-sized 150-μm spheroids can be uniformly absorbed in culture media and inhibit apoptosis, which ensures constant proliferation and differentiation of cells into spheroids. The size distribution of native pancreatic islets ranges from 50 to 500 um [40]. In the field of pancreatic islet transplantation, larger human islets showed an increasing percentage of necrosis when exposed to 24 h of hypoxia in comparison with smaller uniform islets. Necrosis may occur in larger 3-D aggregates due to a poor supply of oxygen and nutrients. In the previous reports, relatively small islets are superior to large ones according to insulin secretion and viability in vitro and in vivo [40,41,42,43]. The ideal size of most well-functioning islets is between 100 and 200 um. In addition, in order to replace damaged organ function, thousands to millions of spheroids would be necessary. Therefore, there is a need to develop methods that simultaneously allow efficient generation of similar ideal-sized spheroids and their large-scale production for approval and clinical application.

Liver cells are a good candidate for reprogramming into DIPCs because they share a common developmental origin with pancreatic cells and are safer than stem cells [23,44,45]. Interestingly, cells transfected with several pancreatic transcription factors showed insulin production [8,9,11]. Despite these efforts, inadequate control of blood glucose levels remains an obstacle for clinical applications. We developed the 3-D DIPC spheroids to mimic a natural islet structure, which maintains cell–cell interactions during differentiation and maturation to overcome the low differentiation efficiency of DIPCs. To compare DIPCs differentiation between culture conditions, we analyzed the mRNA profiles of endocrine hormones and pancreatic transcription factors involved in insulin production. DIPCs and DIPC spheroids showed higher expression of insulin, glucagon, somatostatin, and pancreas-specific transcription factors, including PDX1, ISL1, NEUROD1, MAFA, NGN3, NKX2.2, NKX 6.1, and FOXA2, than that of liver cells. Particularly, insulin mRNA levels were significantly higher in DIPC spheroids than in DIPCs cultured on plates, whereas glucagon mRNA was not increased in spheroids. Similarly, most pancreatic transcription factors related to β-cell differentiation were higher in DIPC spheroids. NEUROD1 and MAFA showed similar patterns in DIPCs and DIPC spheroids because they were transduced by vectors. Interestingly, PDX1 expression was significantly higher in DIPC spheroids than in DIPCs, indicating the induction of both ectopic and endogenous expression. Spheroid formation may increase the maturation of genes induced in trans-differentiated liver cells along the β-cell lineage. The selectivity of β-cell differentiation may increase in spheroids for various reasons. In liver reprogramming, PDX1 not only induces the β-cell lineage, as demonstrated by increased β-cell-specific transcription factor expression, but also activates glucagon gene expression [46]. To increase the efficiency of β-cell differentiation, α-cell differentiation into glucagon-secreting cells should be prevented. NKX6.1 may be detrimental to glucagon expression in β-cells due to protein–protein interactions between PDX1 and PAX6 or competitive binding of NKX6.1 to the PAX6 binding site on the G1 element of the glucagon promoter [47]. Our results revealed dramatically increased NKX6.1 in spheroids compared to single DIPCs. Similarly, insulin production was higher in the spheroid group, as determined by ELISA, immunocytochemistry, and TEM images. These findings demonstrated that the 3-D culture promoted the levels of genes that affect pancreatic β-cell differentiation. Three-dimensional DIPC spheroids showed better insulin expression and reduced glucagon expression compared to 2-D DIPCs. Therefore, the 3-D culture highly induced differentiation to pancreatic β-cells rather than α-cells.

The morphology, size distribution, differentiation function, and viability of the spheroids produced through the two different methods were compared and evaluated to confirm the difference between the concave microwells and suspension culture technique. Both DIPC spheroids made by suspension culture and concave microwell showed a spherical shape. The average diameter of DIPC spheroids from the suspension culture was slightly less than that of spheroids obtained from concave microwells. The size distribution of spheroids made from concave microwells was relatively uniform, but spheroids made from suspension cultures had a very wide size distribution, and some very large aggregates were formed. Such a large size distribution can be a problem because differentiation is not uniformly performed, and there is an insufficient nutrient supply into the large aggregates during long-term culture. Insulin production and cell viability is higher in DIPC spheroids made by microwells than suspension culture, which means a uniform size of the spheroid could improve DIPC function. Inducing differentiation in spheroid formation can improve the differentiation capacity. However, if spheroids of an uneven size are formed, the quality control for clinical application of cell therapy products may be limited.

Studies have reported that 3-D structures promote DIPC differentiation, but the mechanism has not been clearly investigated. We identified genes that were significantly increased or decreased in spheroids using microarray analysis. Interestingly, cytoskeleton-related genes, such as myosin, actin, and TAGLN, were significantly reduced. Recent studies reported that changes in cytoskeleton regulation are involved in the endodermal lineage [48,49]. In pancreatic endocrine differentiation, recently, Hogrebe et al. reported that actin polymerization can affect the endocrine differentiation in iPSC cells [49]. We confirmed this mechanism in adult cell-based differentiation using 3-D spheroids. We found transgelin is a key regulator to change actin polymerization. Transgelin, known as a protein encoded by the *TAGLN* gene, controlled the transformation and shape change of actin in fibroblast and smooth muscles. In our study, we used cells isolated from adult liver, which has mesenchymal characteristics, and induced trans-differentiation from mesoderm to endoderm. Our study is the first to propose that transgelin is an important factor in trans-differentiation from mesoderm to endoderm. To verify these results, we confirmed that DIPC differentiation increased following treatment with drugs that reduce myosin and actin expression in the 2-D culture. Blebbistatin modulates the actin–myosin molecular interaction by inhibiting the binding of myosin to actin [17], and Y-27632 is widely used as a Rho-kinase selective inhibitor to induce loss of stress fibers and decrease contractility. Following drug treatment, insulin gene expression was significantly increased compared to that in the untreated 2-D culture, as confirmed by qPCR and fluorescence imaging. Upon culturing DIPCs on 2-D plates, elongated and strengthened actin filaments could be identified. However, DIPC spheroids showed relaxed cytoskeletal tension with very thin cortical actin filaments outlining the cells. Similarly, the gene expression of actin, myosin, and TAGLN was reduced. Blebbistatin treatment did not affect myosin, significantly reduced actin expression, and increased insulin expression compared to Y-276323, indicating that the decrease in actin was more closely related to DIPC differentiation. These treatments revealed that differentiation could be improved by controlling actin expression in 2-D culture. Figure 4D shows a scheme describing β-cell differentiation-related cytoskeleton interactions that were downregulated in spheroids. Additionally, E-cadherin expression was significantly increased in spheroid culture, which has been demonstrated as being crucial for proper insulin secretion [50,51,52].

We compared changes in blood glucose levels and body weights among diabetic mice that were untreated or in which DIPCs or DIPC spheroids had been transplanted. Only the DIPC spheroid-transplanted group showed a significant decrease in the initial blood glucose level. Four weeks after transplantation, this group still showed significantly decreased blood glucose compared to the diabetic and DIPC-transplanted groups. Body weights did not decrease in DIPCs- or spheroid-transplanted groups but gradually decreased in the control diabetic group. Similarly, histological staining revealed clear positive cells expressing PDX1 and insulin, which were prominent in the spheroid group compared to the DIPC-transplanted group. Generally, cells transplanted in clusters show increased survival compared to individual cells [53,54]. To confirm cell survival, cells were tagged with NIR Qdot and examined using IVIS. Because the depth of organs that can be confirmed by IVIS is limited, DIPCs and DIPC spheroids were implanted in both subcutaneous sites, and NIR images were analyzed. Immediately after transplantation, similar survival rates were observed; however, individual cells rapidly decreased in the early transplantation stage, and <50% of cells remained after one week. Meanwhile, spheroids gradually decreased, with 84.1 + 5.2% remaining after two weeks. Therefore, spheroid formation was confirmed to improve both the function and survival rate of differentiated cells. Based on these results, spheroid formation improved differentiation function in vitro and efficacy in vivo; such an enhancement of function can increase the possibility of clinical application of differentiated insulin cells. However, we still have many hurdles before 3-D DIPC can be put to clinical use. Fabrication of more mature forms of DIPCs, standardization of induction techniques and a proper source of stem cells, mass production for human scale, and quality control standardization are crucial steps before clinical application. In addition, immune protection of DIPC implantation should also be overcome for widespread clinical application.

## 5. Conclusions

Our results demonstrate the large-scale production of fabricated 3-D DIPC spheroids of the desired shape and size using concave microwells. Moreover, 3-D spheroid formation enhanced the differentiation efficacy and function of DIPCs in vitro and in vivo through actin–myosin-associated cytoskeleton change. This strategy can be translated to the clinical application.

## Supplementary Materials

The following are available online at https://www.mdpi.com/2073-4409/9/12/2551/s1, Figure S1. Ectopic expression of the transduced genes was optimized by testing in various 2D and 3D culture conditions, Figure S2. Comparison of properties and differentiation function of DIPC spheroids made by suspension and concave microwell, Table S1: Primers used for qPCR, Table S2. Top upregulated transcripts between DIPCs and DIPC spheroids as determined by microarray analysis, Table S3. Top downregulated transcripts between DIPCs and DIPC spheroids as determined by microarray analysis
