# Supplementary material for: Spheroid Fabrication Using Concave Microwells Enhances the Differentiation Efficacy and Function of Insulin-Producing Cells via Cytoskeletal Changes
Source: Cells. 2020 Nov 27;9(12):2551. doi: 10.3390/cells9122551 (PMC7768489; doi:10.3390/cells9122551)
Supplement: Supplementary file 1 [file cells-09-02551-s001.pdf]

Spheroid fabrication using concave micro-wells enhanced the differentiation efficacy and function of insulin-producing cells via cytoskeletal changes

Yu Na Lee[1], Hye-Jin Yi[1], Hanse Goh, Ji Yoon Park[1,2], Sarah Ferber[3], In Kyong Shim[1,*], Song Cheol Kim[1,4,*]

[1] Asan Institute for Life Sciences, Asan Medical Center, University of Ulsan College of Medicine, Seoul, Republic of Korea

[2] Department of Chemistry, Wesleyan University, Connecticut, United States

[3] Sheba Regenerative Medicine, Stem Cells and Tissue Engineering Center, Sheba Medical Center, Tel-Hashomer, Israel

[4] Department of Surgery, Asan Medical Center, University of Ulsan College of Medicine, Seoul, Republic of Korea

Figure S1. (A) Ectopic expression of the transduced genes was optimized by testing in various 2D and 3D culture conditions. The scheme for gene treatment conditions during spheroid formation is shown. (B) Fluorescence microscopy images and (C) flow cytometric analysis of GFP-transduced DIPCs and DIPC spheroids in various treatment conditions. When spheroids had already formed in the wells, only surface-level cells (36.0±11.1% of cells) were transduced and expressed GFP. However, when the medium and cells were simultaneously mixed with adenoviral vectors in the microwells, >80% cells were transduced and sufficiently expressed ectopic genes.

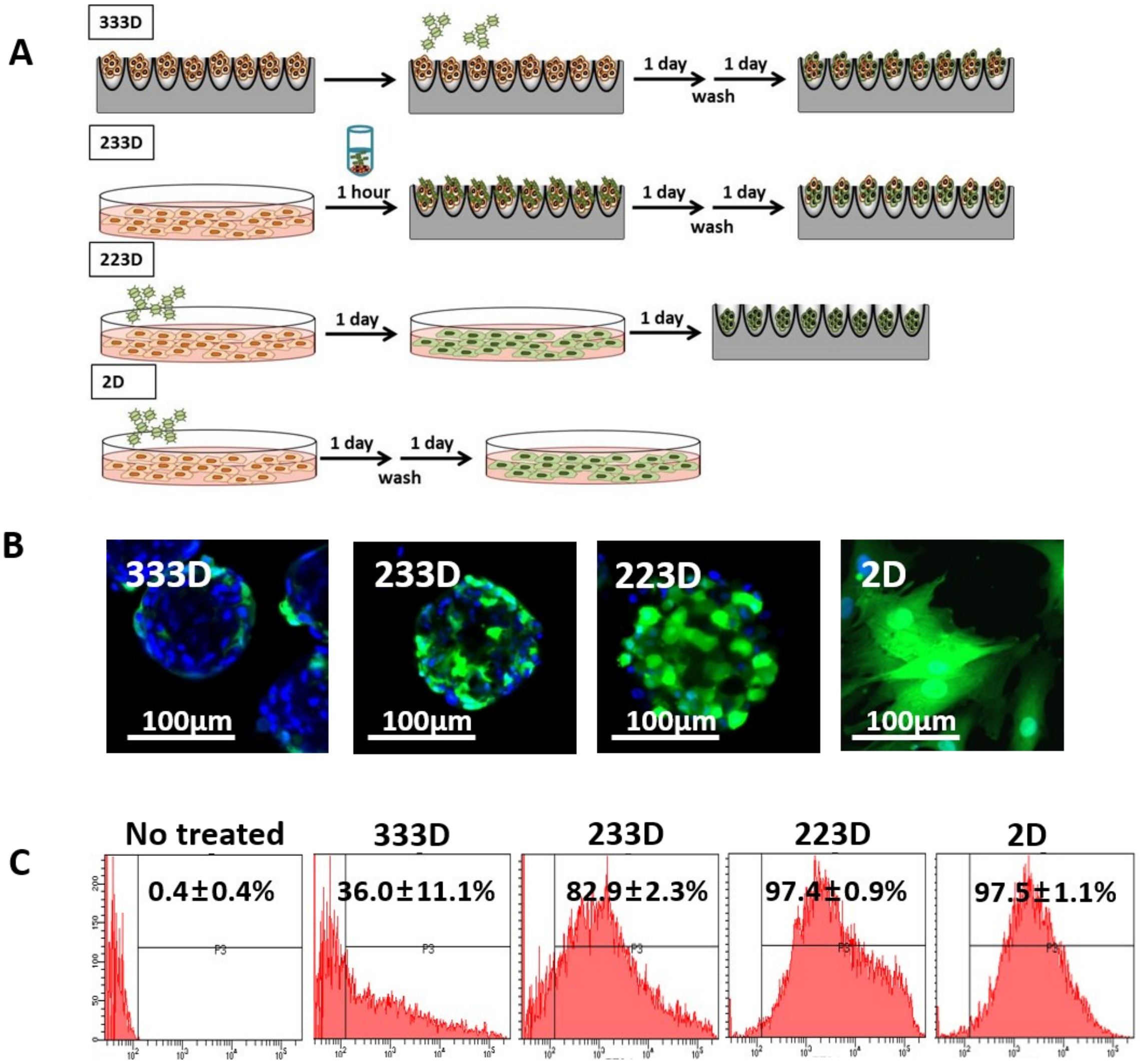

Figure S2. Comparison of properties and differentiation function of DIPC spheroids made by suspension and concave microwell. (A) Representative microscopic images of DIPC spheroids. Both IPC spheroids made by suspension culture and concave microwell showed spherical shape. (B) Size distribution of DIPC spheroids. Average diameter of DIPC spheroids from suspension culture is 152.88+83.98, which is slightly less than that of those obtained from concave microwells (175.62+16.81). The size distribution of spheroids made from concave microwells was relatively uniform, but spheroids made from suspension cultures had a very wide size distribution, and some very large aggregates were formed. (C and D) Gene expression and insulin content on DIPC spheroids compared with liver cells and DIPCs. Both DIPCs made by suspension culture and concave microwell showed similar endocrine gene expression levels and were higher than DIPCs. However, insulin contents of DIPC spheroids made by suspension culture were higher than those of DIPCs but lower than those of microwell DIPC spheroids. (E) Cell viability assay of spheroids with different techniques at day 3 after spheroid formation, using live/dead staining with fluorescein diacetate/propidium iodide (FDA/PI). Viable cells appear as green, while nonviable cells appear as red. In some suspension culture conditions, there were some dead cells in large spheroids, but not many dead cells were found in either condition.

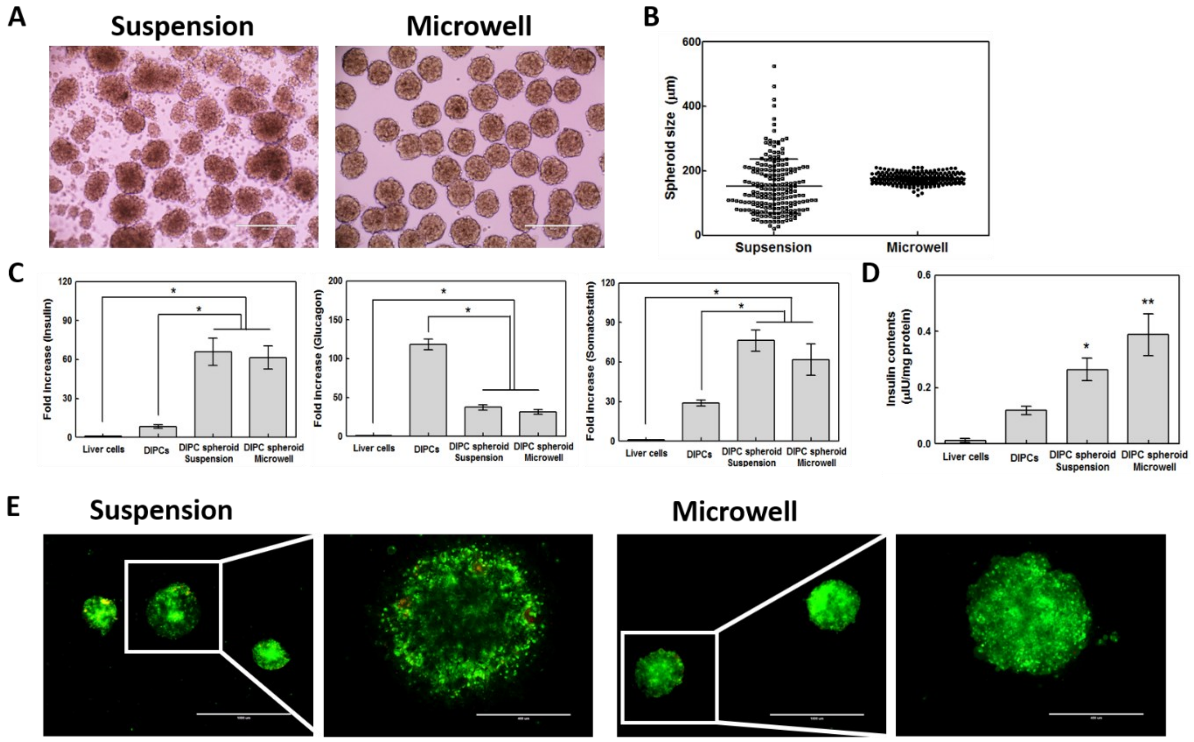

Table S1: Primers used for qPCR

Gene		Sequence (5′→3′)	Product size (bp)
**E-cadherin**	Forward	CGAGAGCTACACGTTCACGG	119
	Reverse	GGGTGTCGAGGGAAAAATAGG	
**FOXA2**	Forward	GCCACCCCAAGACCTACAG	162
	Reverse	GGTTCAGCCGGTAGAAGGG	
**GAPDH**	Forward	GAAGGTGAAGGTCGGAGT	226
	Reverse	GAAGATGGTGATGGGATTTC	
**Glucagon**	Forward	CCCAAGATTTTGTGCAGTGGTT	221
	Reverse	GCGGCCAAGTTCTTCAACAAT	
**GLUT2**	Forward	AGCTTTGCAGTTGGTGGAAT	300
	Reverse	AATAACAATGCCCGTGACGA	
**Insulin**	Forward	GCAGCCTTTGTGAACCAACAC	67
	Reverse	CCCCGCACACTAGGTAGAGA	
**ISL1**	Forward	ATTTCCCTATGTGTTGGTTGCG	229
	Reverse	CGTTCTTGCTGAAGCCGATG	
**MAFA**	Forward	TTCAGCAAGGAGGAGGTCAT	216
	Reverse	CGCCAGCTTCTCGTATTTCT	
**M-cadherin**	Forward	CGTCAGCGGAGTGGATGTG	153
	Reverse	GACAGAACACCCTCGTTGGT	
**MYH9**	Forward	GGGCACTGTCAAGTCCAAGT	153
	Reverse	AGCAGCACATCCTTCAGCTT	
**MYH10**	Forward	GCAGAACAAGGAGCTGAAGG	164
	Reverse	TGCGACGGACTAATTTGTTG	
**NEUROD1**	Forward	CCCTGTACACCCCTACTCCT	92
	Reverse	GAGGCTTAACGTGGAAGACA	
**NGN3**	Forward	GAAAGGACCTGTCTGTCGCT	124
	Reverse	AGGGAGAAGCAGAAGGAACA	
**NKX6.1**	Forward	CACACGAGACCCACTTTTTC	76
	Reverse	CCGCCAAGTATTTTGTTTCT	
**PDX1**	Forward	GCATCCCAGGTCTGTCTTCT	140
	Reverse	CACTGCCAGAAAGGTTTGAA	
**Somatostatin**	Forward	CTGTCTGAACCCAACCAGAC	90
	Reverse	CAGCTCAAGCCTCATTTCAT	

Table S2. Top upregulated transcripts between DIPCs and DIPC spheroids as determined by microarray analysis

Gene symbol	GenBank Accession No.	Description	Fold change
PSTK	NM_153336	phosphoseryl-tRNA kinase	577.95
SLC9A7	NM_001257291	solute carrier family 9, subfamily A (NHE7, cation proton antiporter 7), member 7	159.78
GDF7	NM_182828	growth differentiation factor 7	111.18
LOC729732	NR_047662	uncharacterized LOC729732	101.59
MEG8	NR_024149	maternally expressed 8 (non-protein coding)	76.65
DRAM1	NM_018370	DNA-damage regulated autophagy modulator 1	62.67
CPEB2	NM_182485	cytoplasmic polyadenylation element binding protein 2	50.90
SNORD7	NR_003037	small nucleolar RNA, C/D box 7	42.93
CA12	NM_001218	carbonic anhydrase XII	40.74
LRRC27	NM_001143759	leucine rich repeat containing 27	15.24
NSDHL	NM_001129765	NAD(P) dependent steroid dehydrogenase-like	14.80
ERCC2	NM_000400	excision repair cross-complementation group 2	12.34
EXOSC5	NM_020158	exosome component 5	10.33
RBM14	NM_006328	RNA binding motif protein 14	6.93
BATF	NM_006399	basic leucine zipper transcription factor, ATF-like	5.73
PDK4	NM_002612	pyruvate dehydrogenase kinase, isozyme 4	5.17

Table S3. Top downregulated transcripts between DIPCs and DIPC spheroids as determined by microarray analysis

Gene symbol	GenBank Accesion No.	Description	Fold change
MYLK	NM_053025	myosin light chain kinase	-33.85
MYLK	NM_053025	myosin light chain kinase	-25.98
TAGLN	NM_001001522	transgelin	-25.87
MYH11	NM_001040114	myosin, heavy chain 11, smooth muscle	-25.49
TAGLN	NM_001001522	transgelin	-23.80
MYH11	NM_001040113	myosin, heavy chain 11, smooth muscle	-22.67
ACAN	NM_013227	aggrecan	-20.99
GREM1	NM_013372	gremlin 1, DAN family BMP antagonist	-16.44
SERPINE1	NM_000602	serpin peptidase inhibitor, clade E (nexin, plasminogen activator inhibitor type 1), member 1	-16.29
CNN1	NM_001299	calponin 1, basic, smooth muscle	-15.97
GREM1	NM_001191323	gremlin 1, DAN family BMP antagonist	-15.53
NRXN3	NM_004796	neurexin 3	-14.82
WFDC21P	NR_030732	WAP four-disulfide core domain 21, pseudogene	-13.74
NSG1	NM_001287763	neuron specific gene family member 1	-13.37
ACTA2	NM_001613	actin, alpha 2, smooth muscle, aorta	-13.15
TXNIP	NM_006472	thioredoxin interacting protein	-13.05
OLFML3	NM_020190	olfactomedin-like 3	-13.02
ACTBL2	NM_001017992	actin, beta-like 2	-12.26
RCAN1	NM_004414	regulator of calcineurin 1	-12.04
F2RL2	NM_004101	coagulation factor II (thrombin) receptor-like 2	-11.95
BIRC5	NM_001012271	baculoviral IAP repeat containing 5	-11.89
ACTG2	NM_001615	actin, gamma 2, smooth muscle, enteric	-11.83
MYL2	NM_000432	myosin, light chain 2, regulatory, cardiac, slow	-11.19
